# Effect of HTST Thermal Treatments on End-Use Quality Characteristics of Goat Milk

**DOI:** 10.1155/2019/1801724

**Published:** 2019-12-02

**Authors:** B. D. Rohitha Prasantha, K. M. S. Wimalasiri

**Affiliations:** Department of Food Science & Technology, Faculty of Agriculture, University of Peradeniya, Peradeniya 2040, Sri Lanka

## Abstract

Goat milk samples were pasteurized at high-temperature (72°C, 75°C, and 81°C) and in short-time (15 s and 25 s) combinations. Physical, chemical and microbial qualities of the pasteurized milk samples were evaluated 0, 2, 3, and 4 weeks of storage at 4°C. Despite the different thermal treatments, specific-gravity and viscosity were comparatively stable immediately after pasteurization (IAP). The viscosity of pasteurized milk at 81°C showed significant increase (*P* < 0.05) from 1.58 ± 0.18 to 2.30 ± 0.15 mPa s during four weeks of storage. Relative lightness “*L*” value decreased by about 10% during the storage period of 81°C pasteurization samples. Acidity increased with heat treatment irrespective of holding time, but in 81°C pasteurized sample higher acidity was developed at end of the storage. Fat oxidation 2-3 times higher at 81°C than 72°C pasteurized samples. Total protein (TP%) and nonprotein nitrogen contents were stable IAP but TP reduced significantly (*P* < 0.05) at two weeks storage. The whey protein denaturation increased with pasteurization treatments and storage time. Antioxidant activity of raw goat milk was 34.8 ± 5.01 *μ*mol l^−1^ and was decreased by 20–43% IAP compared to raw milk samples, but gradually increased during storage. IAP, mesophilic counts were in the range of 980–110 cfu ml^−1^ (72°C/15–81°C/25 s) and increased from 2236 to 680 cfu ml^−1^ samples stored at 4 weeks. Results showed that best quality stability of pasteurized goat milk achieved by heat treatments between 72°C/25 s and 75°C/25 s heat treatments up to 3 weeks of storage under 4°C.

## 1. Introduction

The fresh milk consumptions in Sri Lanka have increased over the last two decades. Average per capita consumption of milk and milk products is about 76 g day^−1^ for an urban person while average rural per capita consumption is about 48 g day^−1^. Full-cream milk powders play an important role in the domestic market, accounting for nearly 60% while liquid milk, curd, yoghurt, ice-cream, and another dairy products account for another 40%. Due to many food safety issues of powdered milk, Sri Lankan government has implemented several national projects to promote the liquid milk consumption in the country since 2010. As a result of that, pasteurized and sterilized cow's milk is now commonly available in the Sri Lankan market. Precise thermal treatments (temperature-holding time) are crucial for prolonged shelf-life and consumer-safety of processed milk. Although many countries have regularized their legislative standards of thermal treatments for pasteurized milk, in Sri Lanka different milk processing-plants use various thermal treatments [1]. However, some physico-chemical characteristics and microbiological quality parameters of raw and processed bovine milk have been specified by the Sri Lanka Standards Institute [2] apart from the quality standards of goat and sheep milk.

There is growing interest and demand for goat milk as a substitute for cow's milk from those who suffer from allergies and intolerance for other milk. Goat milk differs from cow or human milk because it has better digestibility, buffering properties and certain therapeutic values in relation to human nutrition [3]. Goat milk contains 8.85% solid nonfat (SNF) and 4.0% milk fat which is more or less similar to the bovine milk which contains 8.25% SNF and 3.25% milk fat. Many developing countries raise goats in small to medium size farms, but the majority of the local farms maintain goats under inferior hygienic conditions. Therefore, establishment of appropriate thermal treatment could be the best way to ensure the safety of fresh goat milk with a longer shelf-life and will help to sustain the goat milk industry in the market.

Microbial growths shorten the shelf-life of pasteurized milk by producing undesirable characteristics and change in sensory attributes [4]. Therefore, consumers have faced serious problems of microbial safety and keeping quality of the products. But there are other factors that affect the keeping quality of pasteurized milk such as fresh milk quality, severity of heat treatment, post pasteurization contamination, and storage temperature. The main objective of thermal process is reduction of microbial load in the fresh milk. The influence of thermal treatment on milk composition depends to a great extent on the temperature applied and the time of exposure. In recent years, many dairy products pasteurized at different temperatures >72°C, in order to reduce the risk of heat resistant pathogenic bacteria. Although psychrotropic bacteria are almost killed by the pasteurization temperature/time at 72°C/15 s, some microbial survivors could promote the spoilage of pasteurized milk before the estimated expiry date [5]. On the other hand, heating of milk >72°C induces denaturation and aggregation of whey proteins and casein micelles [6]. Goat milk has very small casein micelles and fat globules than bovine milk [7]. Therefore, high thermal treatments may directly affect the keeping quality of goat milk than cow's milk. This phenomenon could lead to gradual decrease in the quality of pasteurized milk leading to decline of the commercial acceptability of pasteurized goat milk.

Majority of Sri Lankan farms maintain goats under inferior hygienic conditions. Therefore, establishment appropriate thermal treatment could be the best way to ensure the safety of fresh goat milk with a longer shelf-life and will help to start the industrial goat milk processing plants in the country. Previous studies have shown the presence of coliforms and *Listeria monocytogenes* in pasteurized milk processed in Sri Lanka [1, 8]. This situation is due to their survival ability over different pasteurization processes, temperature-time variations or the pasteurization method. Therefore, the occurrence of *L. monocytogenes* and high coliform counts in raw milk is the most common scenario in small scale goat farms in Sri Lanka. As a consequence of that significant control of mesophilic and psychrotrophic counts in milk prior to consumption is vital to ensure the safety of fresh milk. Above information indicates the requirement of finding the effect of different pasteurization thermal treatments on the goat milk quality and to establish the optimum pasteurization conditions. Therefore, the objective of the present study was to determine the influence of pasteurized thermal treatment conditions and storage time on the physical, nutritional, antioxidant, and microbiological characteristics of HTST pasteurized goat milk.

## 2. Materials and Methods

### 2.1. Sample Collection and Pasteurization

Raw goat milk (fresh milk) samples (5 L sample) were purchased from a four small-holder goat farm (indigenous goat breed “Kottukachchiya”). Milk samples were immediately transported to the laboratory (Department of Food Science and Technology) in a sterilized stainless-steel tank at ambient temperature (≈25°C) within 30 min. All 4 samples were mixed to obtained whole bulk sample. Pasteurization was carried out immediately after receiving of raw goat milk, using high temperature short time (HTST) process. The HTST treatment was carried out in a laboratory stainless steel plate heat exchanger (Armfield-FT43A, USA). The milk sample was stirred and introduced into the system by a peristaltic pump and heat exchanger was setup to the temperature-holding time combinations of 72°C, 75°C, or 81°C for 15 or 25 s. Altogether six heat treatments (72°C/15 s, 72°C/25 s, 75°C/15 s, 75°C/25 s, 81°C/15 s, and 81°C/25 s) were used for pasteurization experiment. The holding time was calculated based on volume of the holding tube (75.8 cm^3^), volume of heat exchanger and flow rate generated by the peristaltic pump. The “come-up time” of pasteurizer was set to less than 2 min. All the pasteurized milk samples were confirmed of their adequacy of HTST process by alkaline phosphatase test [9]. Immediately after pasteurization, the milk sample was shaken and homogenized using an ultrasonic homogenizer (Fisher sonic dismembrator, model 150; USA). Homogenized milk samples were bottled into 350 ml sterilized glass bottles, capped, labeled under sterile condition (Laminar flow cabinet) and stored in a refrigerator at 4°C for further analysis. All the samples were analyzed immediately after pasteurization (IAP), two, three, and four weeks after storage for their physical, chemical, and microbiological qualities.

### 2.2. Physical Properties

The specific gravity was determined according to the stranded method [9] of using specific gravity bottle. Apparent viscosity was measured in coaxial-cylindrical rotational viscometer (Tokimec-B, Japan), equipped with an adapter (BL adapter) for low viscosity and small samples analysis. Color of milk samples were analyzed by measuring the reflectance, using a spectrophotometer (Nippon-ZE 2000, Japan). A glass cell (30 mm diameter) containing the sample was placed in the instrument and the “*L*” value was measured in triplicate to describe the color change in relative lightness of the pasteurized milk samples.

### 2.3. Protein, Casein and Whey Protein Denaturation

Total nitrogen contents (w/w%) and casein nitrogen contents (w/w%) of the milk samples were determined by using Kjeldahl method and sodium acetate-acetic acid precipitation method respectively. The nitrogen contents were then converted to protein multiplying by 6.38 as the conversion factor from nitrogen to total protein (TP%) and casein protein (CP%) contents. Nonprotein nitrogen (NPN% w/w) was determined by solubility in tri-chloroacetic acid (12% w/v) [9, 10]. The whey protein (WP%) in raw and pasteurized milk was calculated using Equation (1). The whey protein denatured (WPD) on heating, as a percentage of total whey protein, was calculated using equation (2).

(1) $$\begin{array}{c} \text{WP}\;\left(\%\right)=\frac{\left(\text{TP}-\text{CP}-\text{NPN}\right)}{\text{TP}}\times 100, \end{array}$$

(2) $$\begin{array}{c} \text{WPD}\;\left(\%\right)=\frac{\left({\text{WP}}_{\text{fm}}-{\text{WP}}_{\text{pm}}\right)}{{\text{WP}}_{\text{fm}}}\times 100, \end{array}$$

where CP, TP, NPN, WP, WPD, WP_fm_, and WP_pm_ are the levels of casein protein contents (w/w%), total protein contents (w/w%), nonprotein nitrogen (w/w%), whey protein (w/w% of TP), whey protein denatured (%), whey protein in the raw milk (w/w%) and whey protein in pasteurized milk, respectively.

### 2.4. Fat Oxidation and Acidity of Goat Milk

Fat oxidation was determined spectro-photometrically using the 2-thiobarbituric acid (TBA) test as described by King [11]. Formation of red pigment during the reaction between 2-thiobarbituric acid and oxidized lipids was measured at 538 nm wave length using a UV-Visible spectro-photometer (1201-Shimadzu; Japan). The TBA value was expressed as milligrams of malonaldehyde per kilogram of milk. Titrimetric method [9] was used to determine the titratable acidity in milk samples. Acidity number was expressed as a percentage of lactic acid content.

### 2.5. Total Fat Percentage

Total free fat was determined by “Gerber” method [2, 9], where milk fat is separated from proteins by adding sulphuric acid. The separation is facilitated by using amyl-alcohol and centrifugation. The fat content was estimated directly using a calibrated butyrometer.

### 2.6. Antioxidant Activity

The free radical scavenging activity of goat milk samples were measured by the 2,2-diphenyl-1-picrylhydrazyl (DPPH) methods described by [12] with some modification. Accurately measured 100 *μ*l of each milk sample and it was mixed with 2.00 ml of DPPH in ethanol solution of 100 mM. The solution was incubated in laboratory incubator at 35 ± 1°C for about 30 min. Incubated samples were centrifuged at 3000×*g* for 10 min., after adding one milliliter of chloroform. The absorbance of the supernatant was determined at 517 nm using a UV–Visible spectro-photometer (1201-Shimadzu; Japan). Absorbance of control samples were measured in parallel to the milk samples. The percentage of DPPH radical scavenging activity was calculated using following Equation (3).

(3) $$\begin{array}{c} \text{SA}=\left[\frac{\left(\text{CA}-\text{MA}\right)}{\text{CA}}\right]\times 100, \end{array}$$

where SA, CA, and MA radical scavenging activity (% or *µ*mol l^−1^), absorbance of the control and absorbance of the sample respectively.

### 2.7. Microbiological Analysis

Standard plate counts were conducted to determine Mesophilic counts in the treated milk samples according to the [13] standard, where plate count agar was used as the growth medium maintained at 30°C for 24 h incubation period before colony count. Psychrotrophic counts were determined according to the [14] where plate count agar was used as the growth medium maintained at 7°C for 2 weeks incubation before taking the colony count. Existence of *L. monocytogenes* (most common in raw milk of Sri Lanka) in milk samples were verified using Listeria-enrichment broth. Test tubes counting inoculated broths were maintained <30°C for 24 h before observing for colonies [13]. Coliform counts were determined using violet red bile agar as the growing medium. Inoculated samples were incubated at 37°C for 48 h before taking the colony count.

### 2.8. Statistical Analysis

All laboratory experiments were performed in triplicate. Chemical compositions and microbial analysis were presented as mean ± SD (standard deviation). Two-factor factorial completely randomized design (3 temperatures × 2 holding times) with repeated measures by storage time was used to monitor the effects of temperature, holding time and their interaction by storage period of the pasteurized milk samples. The data were subjected to two-way analysis of variance (ANOVA) and means were determined using the Duncan's multiple range test at significant level *P* < 0.05 using SAS software.

## 3. Results and Discussion

### 3.1. Specific Gravity and Viscosity

**Figure 1:**
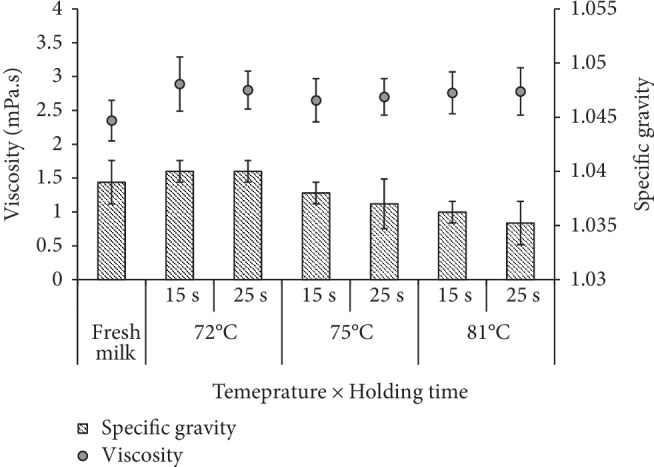
The effect of heat treatments on the viscosity and the specific gravity of goat milk immediately after pasteurization (IAP) at 72°C, 75°C, and 81°C temperature and holding times of 15 and 25 s.

There was no significant difference (*P* > 0.05) among specific gravity values (SG) of raw milk and different heat treatments (Figure 1) except decreasing trend was shown with increasing temperature. Moreover, SG did not change significantly (*P* > 0.05) within the storage time with respect to the heat treatments (temperatures/holding times). The SG values were in the range of 1.039 ± 0.001–1.042 ± 0.005 at storage temperature of 4°C. Despite pasteurization temperature, SG values had no significant difference (*P* > 0.05) between two different holding times of 15 and 25 s. Average SG values of two different holding times were 1.041 ± 0.003, 1.039 ± 0.001, and 1.038 ± 0.001 for 72°C, 75°C, and 81°C pasteurization, respectively. Viscosity has also followed similar behavior like SG and no significant differences (*P* > 0.05) were observed within the different heat treatments.

Raw goat milk showed viscosity of 1.62 ± 0.2 mPa s (Table 1) but it was slightly increased IAP without significant change (*P* > 0.05) to the viscosity values. Average viscosity values were given as 1.71 ± 0.17, 1.67 ± 0.14, and 1.58 ± 0.18 mPa s for pasteurization temperatures of 72°C, 75°C, and 81°C, respectively. The reason for change of SG and viscosity IAP is denaturation of whey protein or shear thinning of product at the initial stage of heating at high heat treatments [15]. Although the average viscosity of 75°C and 72°C pasteurized milk samples did not change significantly (*P* > 0.05) with the storage time, viscosity of milk pasteurized at 81°C increased significantly (*P* < 0.05) from 1.58 ± 0.18 mPa s to 2.30 ± 0.15 mPa s during the four weeks of storage at 4°C (Table 2). This could be due to the damage fat globules of milk, denaturation of milk protein during the heat treatment and later gradual separation of cream layer during storage [15, 16] at low temperature while limiting the shelf-life.

Swelling of casein micelles mainly caused by acidity changes of the milk and that may later result in increased viscosity of stored goat milk pasteurized at 81°C [16]. The other reason of viscosity increase during storage, may be attributed to the initiation of age gelation process of milk protein due to interaction between whey protein and casein at high thermal treatment [15–17]. Therefore, high thermal treatments (>81°C/15 s) may directly affect the viscosity of stored goat milk while limiting the shelf-life <3 weeks.

### 3.2. Milk Color

**Figure 2:**
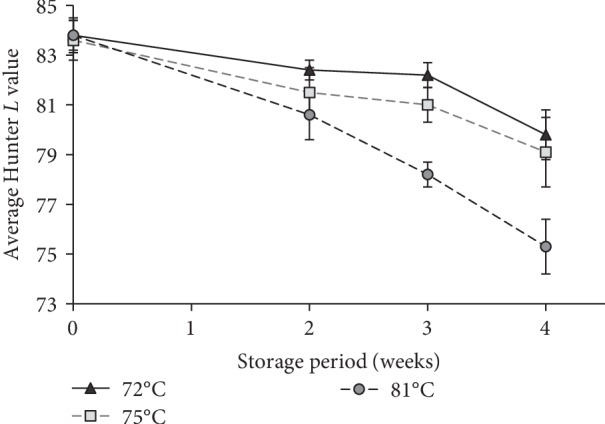
The effect of average pasteurization temperature (time × temperature) on the Hunter color value “*L*” or relative lightness of pasturized goat milk storge at the temperature of 4°C.

Hunter color value “*L*” was used to analyze the relative lightness of the raw, pasteurized and stored goat milk samples. Irrespective of pasteurization time, with the storage, *L* value gradually decreased but the reduction was significantly high (*P* < 0.05) within the 81°C (Figure 2) than other two pasteurization temperatures. The highest *L* value of 83.8 ± 0.4 was observed in raw milk (Table 1) and IAP. The lowest *L* value of 75.3 ± 1.1 was observed in the 4 weeks stored samples pasteurized at 81°C. However, the difference between the highest (IAP) and the lowest *L* (81°C) value was only 10.1% which may not significantly affect appearance of the stored samples but affect other sensory characteristic (data not present here).

The *L* values of raw and pasteurized milk in the present study were more or less similar to the previously reported data [18, 19]. The change in *L* value of pasteurized goat milk during storage may be due to the “Maillard” browning reaction, formation of various other organic compounds during thermal degradation [19] and further activity of mesophilic bacteria. Reddy Nguyen [20] has reported that decrease in lightness and development of opacity in UHT treated milk resulted due to the increased viscosity during the storage period similar to this study.

### 3.3. Titratable Acidity

Raw goat milk has an acidity of 0.17 ± 0.01% (Table 1) and it was significantly increased (*P* < 0.05) to 0.22 ± 0.02% IAP at the 81°C, but subsequently reduced to an average of 0.18 ± 0.02% during 2 weeks of storage at the 4°C (Table 3). Milk pasteurization between 72 and 75°C did not change significantly (*P* > 0.05) their titratable acidity IAP but showed tentative increase of acidity. Degradation of lactose at high heat treatments >75°C might be the reason of increased acidity IAP at 81°C of heat treatment [21, 22]. However, acidity of milk pasteurized at 81°C increased to 0.22 ± 0.02% at the end of storage period of 4 weeks whereas milk acidity of other samples did not change even after 4 weeks of storage at low temperature. Whey protein denaturation and interaction between lactose and protein, or formation of organic acids may be the reason for the subsequent increase in acidity of pasteurized milk 81°C during storage. The other reason for acidity development IAP and during storage may be correlated to the initial high mesophilic load and loss of acid buffering capacity of milk because of protein denaturation at high heat treatments. High mesophilic count may degrade the lactose sugar present in milk and gradually acidify the sample during storage while reducing the milk stability [7]. In this study, we observed gradual increase of milk acidity during storage to indicate that goat milk is less stable to higher thermal treatments.

### 3.4. Percentage of Total Fat

**Figure 3:**
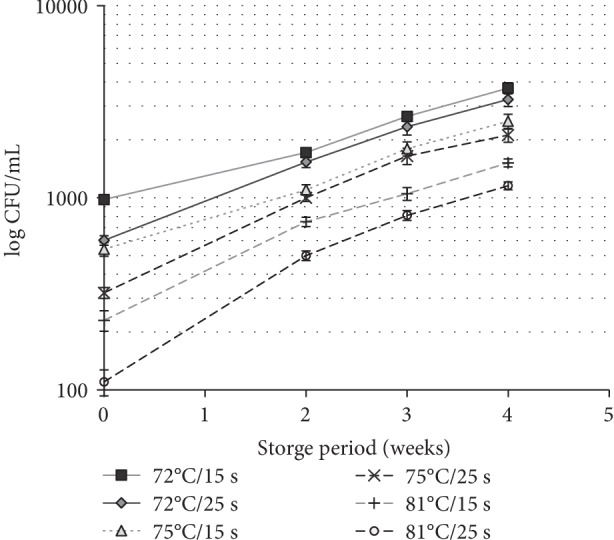
Mesophilic bacteria count (log CUF ml^−1^) of pasteurized goat milk stored at 4°C. Temperature–time combinations of 72°C/15 s, 72°C/25 s, 75°C/15 s, 75°C/25 s, 81°C/15 s, and 81°C/25 s considered as heat treatments.

Total fat content of raw goat milk was 4.1 ± 0.2% (Table 1). Relatively small amount of milk fat has changed (*P* > 0.05) in the milk samples IAP (Table 2). Pasteurization at 72°C and 75°C showed very little effect (*P* > 0.05) on breakdown of fat compared to 81°C. However, milk fat decreased significantly (*P* < 0.05) during the storage at 4°C. After 4 weeks of storage, about 18% of fat reduction was observed in the pasteurization milk sample at the 81°C. Previous study found that fat content of stored cheese decreased with increasing pasteurization from 72–87°C [21]. The reduction of fat with respect to the higher heat treatments may be associated with some change in the protein membrane of milk fat globule because goat milk has smaller fat globules than bovine milk [7]. Therefore, reductions of fat content in goat milk with increasing heat treatments related to the indefinite change the nature of fat globules and/or enhance lipase activity due to mesophilic activity of stored milk (Figure 3). In contrast to that, Garcia et al. [23] have reported that fat content, viscosity and density of goat milk showed minimal sensitivity to the pasteurization of 72°C, 76°C, and 80°C with holding times of 15 s and 10 s. This study revealed that fat content and viscosity of pasteurized goat milk were sensitive to the pasteurization temperature 72–81°C than holding time of 15–25 s. Fat content of the tested goat milk samples showed higher values than the bovine fat content of the Sri Lanka standards [2].

### 3.5. Fat Oxidation

The 2-thiobarbituric acid (TBA) values indicate the intensity of autoxidative degradation of fats (mg of malonaldehyde kg^−1^ of milk) of raw milk and stored pasteurized milk (Table 3). The TBA value of raw goat milk sample was 0.007 ± 0.004 mg of malonaldehyde kg^−1^ of milk (Table 1). A significant change (*P* < 0.05) in the TBA values was noted between 3-4 weeks of storage at 4°C. During 3 weeks of storage, TBA values increased to their maximum of 0.16 ± 0.01 mg of malonaldehyde kg^−1^ of milk at the 81°C. That was about 95% increase of fat oxidation compared to the raw milk sample. The intensity of fat oxidation at 81°C was 2-3 times higher than the lowest pasteurization treatment temperature of 72°C/ 15 s. Smet et al. [24] reported that during storage of dairy products, primarily oxidized products were formed as a function of time. The results of this study also showed similar behavior in relation to the fat oxidation of pasteurized milk storage 4 weeks at chilled condition. These changes may lower the acceptable quality of pasteurized goat milk after 3 weeks of storage at 4°C. Zygoura et al. [25] found that TBA value gradually increased among pasteurized milk during 7 days of storage at 4°C. Panfil-Kuncewicz et al. [26] noted the TBA value of UHT milk increased constantly during 6 months of storage at 4°C. The results of this study were in agreement the severity and types of the heat treatments which boost the fat oxidation of pasteurized goat milk.

### 3.6. Total Protein and Nonprotein Nitrogen

**Figure 4:**
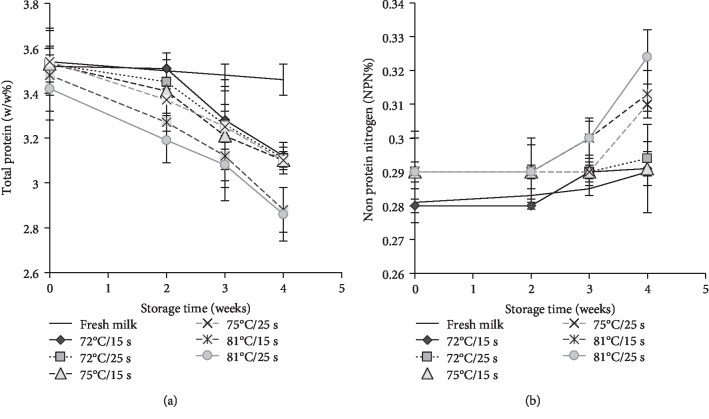
Changes of percentage of total protein (a) and nonprotein nitrogen (b) contents of pasturized goat milk storge at the temeprature of 4°C. Temperature time combinations of 72°C/15 s, 72°C/25 s, 75°C/15 s, 75°C/25 s, 81°C/15 s, and 81°C/25 s are considered as heat treatments.

Percentage of total protein (TP%) and nonprotein nitrogen (NPN%) contents of milk did not change significantly (*P* > 0.05) IAP. The TP content reduced significantly (*P* < 0.05) 2 weeks after storage (Figure 4) but NPN content did not change significantly (*P* > 0.05) throughout the storage period. However, both TP and NPN changed significantly (*P* < 0.05) at 81°C than at other temperatures. It was noted that NPN content increase at higher thermal treatments may be attributed to the breakdown of milk proteins at higher heat treatment or by microbial activity. Goat milk contains about 0.7% of nitrogen which is distributed in fractions of 4.6% TP [7]. However, our data showed lower NPN of 0.28 ± 0.08% and TP of 3.54 ± 0.06% in raw goat milk (Table 1). This could be the differences between species, feeds and climatic condition.

It has been reported that the NPN content of the heat-treated milk did not change if the heat treatment was maintained between 72–80°C [23]. According to our data, there was no significant change of TP and NPN contents IAP but TP contents steadily declined during the prolong storage at 4°C.

### 3.7. Casein Content

Percentage of casein content with respect to the heat treatments is given in the Table 4. Casein of raw milk was 2.65 ± 0.07% (Table 1) but did not change significantly (*P* > 0.05) IAP treatments. Average casein content was 2.64 ± 0.02% IAP but it gradually decreased from 2.48% to 2.32% (72°C/15–81°C/25 s) after 2–4 weeks of storage. According to previous study, casein content of cow milk did not change within the pasteurization temperature between 72–87°C [21]. Results of this study showed drop of TP and casein contents of pasteurized goat milk because of heat treatments and storage time.

### 3.8. Whey Protein

Whey protein content (WP%) in the goat milk changed throughout the pasteurization treatments and storage time (Table 4). Raw milk contained 17.23% WP and it significantly reduced IAP by about 15% at 81°C/25 s heat treatment. However, WP content was comparatively stable throughout the other heat treatments such as 72°C/15 s, 72°C/25 s, and 75°C/15 s. The percentage of whey protein denaturation (WPD%) was also increased as the heat treatments increased from 72°C/15 s to 81°C/25 s with the storage time. According to the results, WPD was significantly increased (*P* < 0.05) from 1.6% to 13.5% IAP with respect to the heat treatments increase from 75°C/ 25 s to 81°C/ 25 s, respectively. After 4 weeks of storage, WPD was significantly increased (*P* < 0.05) in a range of 28.7–82.0% within the heat treatments of 72°C/15 s–81°C/25 s. Irrespective of holding time, significantly higher (*P* < 0.05) WPD was found in the milk samples pasteurized at 81°C compared to 72 and 75°C samples. Previous studies have confirmed that WPD of goat milk is lower than cow milk and it was around 27% at 80°C [22, 23]. Whey proteins are unstable to heat and easily denatured during thermal processing because of unfolding of protein molecule. Increase acidity of pasteurized goat milk at 81°C may also be contributed to the higher WPD% during storage.

### 3.9. Antioxidant Activity

**Figure 5:**
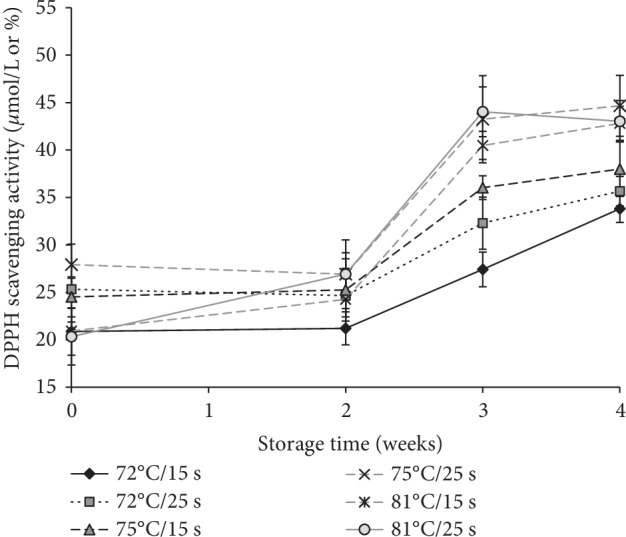
Total antioxidant capacity of pasteurized goat milk as DPPH (2,2-diphenyl-1-picrylhydrazyl) radical scavenging activity (*μ*mol l^−1^) as a function of storage time. Temperature and time combinations of 72°C/15 s, 72°C/25 s, 75°C/15 s, 75°C/25 s, 81°C/15 s, and 81°C/25 s are considered as heat treatments.

Total antioxidative activity (AOA) of goat milk was indicated by the 2,2-diphenyl-1-picrylhydrazyl (DPPH) scavenging activity (Figure 5). The DPPH radical scavenging activity or AOA of raw goat milk was 34.8 ± 5.01 *μ*mol l^−1^. The AOA of pasteurized milk decreased by about 20–43% IAP compared to the raw milk samples. Despite the importance of holding times, the AOA pasteurized milk significantly increased (*P* < 0.05) by 38–57% between 72–81°C treatments 2 weeks after storage at 4°C.

The highest AOA was observed in the 81°C pasteurized and stored goat milk than 72°C and 75°C heat treatments. During the first two weeks of storage, the AOA of the goat milk was nearly constant, probably because of stable acidity content (Table 3) or neutralization free of radicals. During heating, milk liberates S‒H group from proteins, which increased the antioxidant activity of the milk but may develop a “cooked test” [22]. Our sensory studies revealed (our unpublished data) “cook/scorching test” development in milk IAP at 81°C/25 s. DPPH scavenging activity of whey protein was much higher than caseins in milk because lactoferrin acts as the key constituent in the whey fraction [27]. According to the results, WPD was increased 2-3 weeks after storage while increasing the AOA of stored goat milk after 2 weeks. The formation of peptides derivatives from breakdown of caseins and whey proteins has high potential to show AOA then other derivative [28, 29]. Klimczak et al. [30] reported that DPPH radical scavenging activity increased upon the storage of orange juice due to the destruction of ascorbic acid. Therefore, gradual break down of heat labile vitamins in milk (i.e. vitamins B and C), denaturation of protein, slow activity of residual enzyme and change of milk acidity during storage may be the reasons for increase of AOA of stored pasteurized goat milk.

### 3.10. Microbiological Characteristics

According to the results, high mesophilic count (≈5.69 log cfu ml^−1^) were detected but low psychrotrophic count (<2.3 log cfu ml^−1^) were found in the raw milk samples (Table 1). However, Listeria and coliform counts were not found in the raw milk. According to the Sri Lankan standard Coliform should be absent in pasteurized bovine milk [2]. Absence of coliform bacteria indicated satisfactory hygienic condition maintained at the goat farm especially during the milk harvesting and also absence of post-pasteurization contamination of milk. Figure 3 indicates the mesophilic counts of pasteurized goat milk stored at 4°C. IAP, mesophilic counts were significantly dropped in the range of 2.99–204 log cfu ml^−1^ (72°C/15 s–81°C/25 s) depending on the temperature and holding time, but no another psychrotrophic counts were detected in the pasteurized samples. Remaining mesophilic bacteria in the IAP samples could be related to the presence of thermal resistant mesophilic bacteria in the raw milk sample. Significant increase (*P* < 0.05) of mesophilic count was observed in the pasteurized milk samples during 4 weeks of storage at 4°C. After 4^th^ week storage at 4°C, mesophilic counts increased from 3820 to 1155 cfu ml^−1^ (3.57–3.06 log cuf ml^−1^) in pasteurized milk samples heat between 72°C/15 s and 81°C/25 s. Craven and Macauley [5] reported maximum accepted levels of mesophilic counts was 7 log cfu ml^−1^ in the pasteurized milk. This study indicated higher microbiological stability of pasteurized goat milk, since the mesophilic population in the milk did not reach 4.0 log CUF ml^−1^ even after 4 weeks of storage at 4°C. Sepulveda et al. [31] found mesophilic counts approximately 4 log cfu ml^−1^ of pasteurized milk at 72°C/15 s after 40 days of storage at 4°C, which was lower microbial count than this study after 4 weeks of storage.

Goat milk has comparatively high AOA and antimicrobial activity due to lactoferrin content [29] which may be the reason for prolonged stability of the pasteurized goat milk. These results indicated that appropriate heat treatments can increased the quality of goat milk, while reducing the mesophilic and psychrotrophic counts and get longer microbiological stability of the pasteurized goat milk. However, milk with higher initial microbial counts produce more heat resistance enzymes which are susceptible to high gel formation and brake down of fat during storage [17, 26] similar to finding of this study. Most importantly, microbial count was comparatively low in pasteurized goat milk even after 4 weeks of storage at 4°C. Therefore, further investigation is necessary to find out the high keeping quality of pasteurized goat milk. The possible reasons may be antioxidant and antibacterial properties of goat milk.

## 4. Conclusion

The results of this study showed that physical, chemical and microbiological changes occurred in HTST pasteurized goat milk when storage under refrigerated temperature of 4°C. The changes lower the product quality and can lead to decline in the commercial acceptability of pasteurized goat milk. It was found that the range and the intensity of quality changes in the pasteurized goat milk was dependent on pasteurization temperature and the storage period rather than holding times. This study concluded that heat treatments ranged from 72°C/25–75°C/25 s had no effect on the physico-chemical, nutritional, antioxidant and microbiological quality characteristics of pasteurized goat milk, which lasted 2-3 weeks under storage at 4°C. Although 81°C showed better preservation effect, other keeping qualities of goat milk were significantly affected due to high heat treatments.

## Figures and Tables

**Table 1:** Physico-chemical characteristics of raw of goat milk.

Parameter	Values of raw milk (±SD)
Specific gravity	1.04 ± 0.002
Viscosity (mPa s)	1.62 ± 0.2
Hunter *L* value	83.79 ± 0.36
Titratable acidity (lactic acid%)	0.17 ± 0.01
Total fat content (w/w%)	4.1 ± 0.2
Fat oxidation (mg of malonaldehyde kg^−1^)^∗∗^	0.007 ± 0.004
Total protein (w/w%)	3.54 ± 0.06
Nonprotein nitrogen (NPN w/w%)	0.28 ± 0.08
Casein protein (w/w %)	2.65 ± 0.07
Whey protein (%)^‡^	17.2
Antioxidative activity (*μ*mol l^−1^)^†^	34.8 ± 5.01
Coliform count (log cfu ml^−1^)	Nil
Mesophilic count (log cfu ml^−1^)	≤5.69
Psychrotrophic count (log cfu ml^−1^)	<2.3

^∗∗^Base on the 2-thiobarbituric acid (TBA) test.

^‡^Calculated using equation (1).

^†^Based on the free radical scavenging activity 2,2-diphenyl-1-picrylhydrazyl (DPPH).

**Table 2:** The effect of pasteurization temperature on the average viscosity and titratable acidity (%) of stored goat milk at 4°C.

Storage (weeks)	Average viscosity (mPa s) ± SD^∗∗^			Average titratable acidity (%) ± SD^∗∗^		
	72°C	75°C	81°C	72°C	75°C	81°C
0^∗^	1.71 ± 0.17	1.67 ± 0.14	1.58 ± 0.18	0.18 ± 0.01	0.20 ± 0.03	0.22 ± 0.02
2	1.73 ± 0.22	1.73 ± 0.11	1.77 ± 0.27	0.17 ± 0.02	0.17 ± 0.01	0.18 ± 0.02
3	1.76 ± 0.18	1.80 ± 0.22	2.20 ± 0.18	0.17 ± 0.01	0.17 ± 0.01	0.18 ± 0.03
4	1.80 ± 0.24	1.80 ± 0.30	2.30 ± 0.15	0.17 ± 0.02	0.18 ± 0.02	0.22 ± 0.01

^∗^Immediately after pasteurization (IAP); ^∗∗^Average values at different temperatures 72°C (72°C/15 s and 72°C/25 s), 75°C (75°C/15 s and 75°C/25 s), and 81°C (81°C/15 s and 81°C/25 s).

**Table 3:** The effect of pasteurization temperature on the total fat content (%w/w) and TBA value (mg of malonaldehyde kg^−1^ of milk) of fat oxidation stored goat milk at 4°C.

Storage (weeks)	Average total fat content (%) ± SD^∗∗^			Average TBA^†^ value ± SD^∗∗^ (mg of malonaldehyde kg^−1^ of milk)		
	72°C	75°C	81°C	72°C	75°C	81°C
0^∗^	4.05 ± 0.10	4.0 ± 0.07	3.97 ± 0.12	0.02 ± 0.00	0.04 ± 0.00	0.06 ± 0.00
2	3.94 ± 0.08	3.90 ± 0.40	3.82 ± 0.08	0.06 ± 0.01	0.09 ± 0.01	0.14 ± 0.00
3	3.71 ± 0.12	3.68 ± 0.06	3.51 ± 0.06	0.06 ± 0.01	0.11 ± 0.02	0.16 ± 0.02
4	3.68 ± 0.10	3.64 ± 0.08	3.25 ± 0.08	0.06 ± 0.02	0.12 ± 0.01	0.17 ± 0.01

^∗^Immediately after pasteurization (IAP); ^†^TAB =2-thiobarbituric acid values; ^∗∗^Average values at different temperatures 72°C (72°C/15 s and 72°C/25 s), 75°C (75°C/15 s and 75°C/25 s), and 81°C (81°C/15 s and 81°C/25 s).

**Table 4:** Effect of HTST pasteurization process and storage time (at 4°C) on protein composition (±SD) of goat milk.

ST^$^	Milk	HTST pasteurization treatments (temperature-time)					
	Protein	72°C		75°C		81°C	
		15 s	25 s	15 s	25 s	15 s	25 s
0^∗^	Casein (w/w%)	2.64 ± 0.05	2.64 ± 0.07	2.63 ± 0.14	2.65 ± 0.22	2.64 ± 0.1	2.62 ± 0.52
	Whey protein (TP%)^†^	17.28	17.00	17.28	17.00	15.80	14.91
	WPD (%)^‡^	0	0	0	1.63	8.23	13.45
2	Casein (w/w%)	2.61 ± 0.02	2.59 ± 0.01	2.56 ± 0.03	2.58 ± 0.01	2.48 ± 0.01	2.48 ± 0.00
	Whey protein (TP%)	17.70	16.52	16.42	14.84	15.29	13.17
	WPD (%)	0	4.11	4.70	14.00	11.26	23.60
3	Casein (w/w%)	2.48 ± 0.02	2.5 ± 0.1	2.49 ± 0.1	2.49 ± 0.01	2.45 ± 0.03	2.43 ± 0.02
	Whey protein (TP%)	15.85	14.73	14.46	14.46	13.56	11.94
	WPD (% )	8.00	14.54	16.07	16.07	21.27	30.73
4	Casein (w/w%)	2.48 ± 0.01	2.45 ± 0.01	2.43 ± 0.01	2.45 ± 0.01	2.24 ± 0.02	2.32 ± 0.02
	Whey protein (TP%)	11.50	11.25	11.61	11.00	11.80	8.04
	WPD (%)	33.03	34.68	32.60	36.35	31.48	53.33

^$^ST = Storage time (weeks) at 4°C; ^†^Whey protein (TP%) = Whey protein as % total protein; ^‡^WPD = Whey protein denatured (%); ^∗^0 = immediately after pasteurization (IAP).

## Data Availability

The data used to support this study are available from the corresponding author upon request.
